# 6p22.3 amplification as a biomarker and potential therapeutic target of advanced stage bladder cancer

**DOI:** 10.18632/oncotarget.1485

**Published:** 2013-10-29

**Authors:** He Shen, Carl D. Morrison, Jianmin Zhang, Willie Underwood, Nuo Yang, Costa Frangou, Kevin Eng, Karen Head, Roni J. Bollag, Sravan K. Kavuri, Amyn M. Rojiani, Yingwei Li, Li Yan, Annette Hill, Anna Woloszynska-Read, Jianmin Wang, Song Liu, Donald L. Trump, Johnson S. Candace

**Affiliations:** ^1^ Department of Cancer Genetics, Roswell Park Cancer Institute, Buffalo, NY 14263; ^2^ Department of Pathology, Roswell Park Cancer Institute, Buffalo, NY 14263; ^3^ Department of Urology, Roswell Park Cancer Institute, Buffalo, NY 14263; ^4^ Center for Biostatistics, Roswell Park Cancer Institute, Buffalo, NY 14263; ^5^ Department of Pharmacology & Therapeutics, Roswell Park Cancer Institute, Buffalo, NY 14263; ^6^ Department of Medicine, Roswell Park Cancer Institute, Buffalo, NY 14263; ^7^ Department of Pathology, Georgia Regents University, Augusta, GA

**Keywords:** bladder cancer, chromosome 6p22, FISH, outcome, survival

## Abstract

Genetic and epigenetic alterations have been identified as to contribute directly or indirectly to the generation of transitional cell carcinoma of the urinary bladder (TCC-UB). In a comparative fashion much less is known about copy number alterations in TCC-UB, but it appears that amplification of chromosome 6p22 is one of the most frequent changes. Using fluorescence *in situ* hybridization (FISH) analyses, we evaluated chromosomal 6p22 amplification in a large cohort of bladder cancer patients with complete surgical staging and outcome data. We have also used shRNA knockdown candidate oncogenes in the cell based study. We found that amplification of chromosome 6p22.3 is significantly associated with the muscle-invasive transitional cell carcinoma of the urinary bladder (TCC-UB) (22%) in contrast to superficial TCC-UB (9%) (p=7.2-04). The rate of 6p22.3 amplification in pN>1 patients (32%) is more than twice that in pN0 (16%) patients (p=0.05). Interestingly, we found that 6p22.3 amplification is as twice as high (p=0.0201) in African American (AA) than European American (EA) TCC-UB patients. Moreover, we showed that the expression of some candidate genes (E2F3, CDKAL1 and Sox4) in the 6p22.3 region is highly correlated with the chromosomal amplification. In particular, knockdown of E2F3 inhibits cell proliferation in a 6p22.3-dependent manner, whereas knockdown of CDKAL1 and Sox4 has no effect on cell proliferation. Using gene expression profiling, we further identified some common as well as distinctive subset targets of the E2F3 family members. In summary, our data indicate that E2F3 is a key regulator of cell proliferation in a subset of bladder cancer and the 6p22.3 amplicon is a biomarker of aggressive phenotype in this tumor type.

## INTRODUCTION

Many genetic and epigenetic alterations have been identified as to contribute directly or indirectly to the generation of transitional cell carcinoma of the urinary bladder (TCC-UB). Genetic alterations occurring in low-grade superficial TCC-UB are most frequently caused by activating mutations of proto-oncogenes, of which fibroblast growth factor receptor 3 (FGFR3) and HRAS are most prevalent, with mutations in up to 75% and 30% of the papillary tumors, respectively [1, 2]. Since both these oncogenes activate the RAS/MEK/ERK signaling pathway, they appear to be mutually exclusive [3]. In contrast, the majority of muscle-invasive TCC-UB arises through inactivation of the tumor suppressor pathways of TP53, RB1 or PTEN [1, 4]. These mutations result in genomic instability and an anti-apoptotic phenotype, which enables tumor progression through accumulation of mutations. Other mutations that are observed in both subsets of TCC-UB include mutations of phosphoinositide 3-kinase (PI3K, >10%) and deletion of the tumor suppressor genes Tuberous Sclerosis 1 (TSC1, 10%), Patched (PTCH, 40% LOH), CDKN2A, and Deleted in Bladder Cancer 1 (DBC1, 50%) [5, 6].

In a comparative fashion much less is known about copy number alterations in TCC-UB, but it appears that amplification of chromosome 6p22 is one of the most frequent changes [7, 8]. In the present study, using The Cancer Genome Atlas (TCGA) dataset and cBio Cancer Genomics Portal analysis, we found that chromosome 6p22 was highly amplified in bladder cancer patients (18%) in comparison to other carcinomas. In our cohort of 365 TCC-UB, we found that amplification of chromosome 6p22 was significantly associated with muscle-invasive TCC-UB (22%) in contrast to superficial TCC-UB (9%) (p=7.2-04). The rate of 6p22.3 amplification in pN>1 patients (32%) is more than twice that in pN0 (16%) patients (p=0.05). Interestingly, we found that 6p22 amplification is as twice as high (p=0.0201) in African American (AA) than European American (EA) TCC-UB patients. We further characterized E2F3 as a major cell proliferation effector of 6p22 amplification and identified distinct target genes regulated by the E2F3 family members.

## RESULTS

### Chromosomal 6p22 amplification in TCGA and RPCI bladder cancer patient

**Figure 1:**
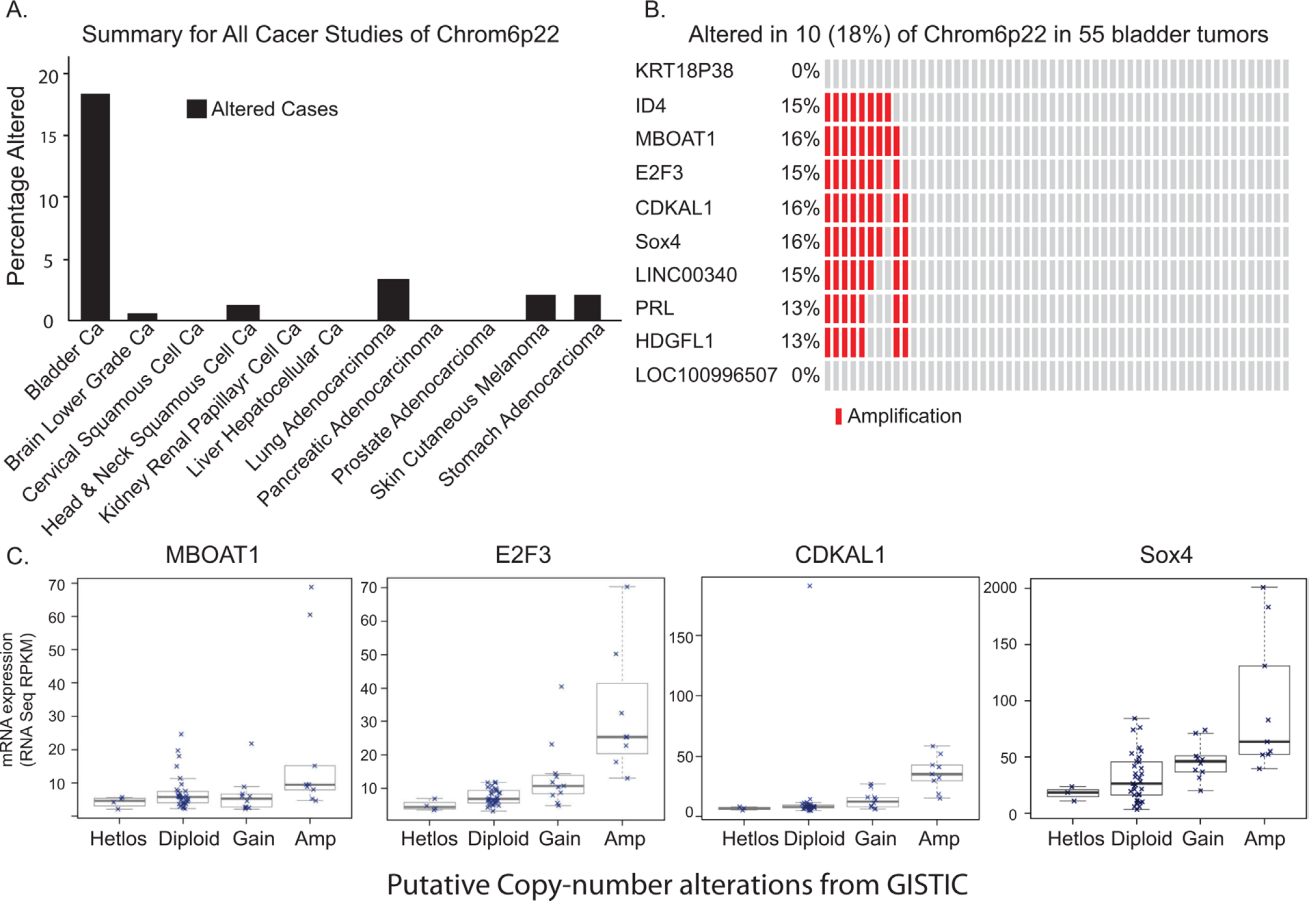
Chromosomal 6p22 amplification in bladder cancer patients A) Data analysis of The Cancer Genome Atlas (TCGA), indicating high prevalence of chromosomal 6p22 amplification in bladder cancer patients. B) High resolution analysis of DNA copy number gains of chromosome 6p22 in bladder cancer in TCGA data. C) Correlation between chromosomal 6p22 amplification and MBOAT1, E2F3, CDKAL1 and Sox4 expression by RNAseq.

With the rapidly declining cost of next-generation sequencing and major national and international efforts such as The Cancer Genome Atlas (TCGA) and the International Cancer Genome Consortium (ICGC) [9], the field of cancer genomics continues to advance at an extraordinarily rapid pace. Using this specific tool, we first examined and identified DNA copy number gains in chromosome 4p16.3, 1p34.2, 12q15, 1q21.3, 10p15.1, 19q12, 8q22.2, 11q13.3, 3p25.2, 1q23.3, and 6p22.3 (Q value above 9.00 E10-3) in bladder cancer patients. We further analyzed the chromosome 6p22 locus across cancer of 11 different origins. To our great interest, the chromosomal 6p22 amplification was highly prevalent in bladder cancer patients (18%) compared to other cancer types (Fig. 1A). Further examination of this region of amplification revealed eight known genes (ID4, MBOAT1, E2F3, CDKAL1, Sox4, LINC00340, PRL, and HDGFL1) (Fig. 1B). Moreover, RNA-seq results showed that CDKAL1, E2F3 and Sox4 are highly expressed in patients with the chromosomal 6p22 amplification (Fig. 1C). To validate this finding in our patients, we performed fluorescent in-situ hybridization (FISH) using RP11 clones from the RPCI bacterial artificial chromosomal (BAC) library in TCC-UB tissue microarrays (TMA) (Supplemental Table 1). Seven different FISH probe sets at 1 to 2 Mb apart spanning the region chr6:17,627,692-25,167,325 were used to define the minimal region of amplification to just over 2Mb as defined by the probe set RP11-239H6 (chr6:20,212,318-20,294,706) and RP11-607F22 (chr6:22,276,349-22,290,957). A complete list of all BAC clones and probe design are listed in Supplementary Table 1 and Supplementary Fig. 1a. A typical positive chromosomal 6p22 amplification FISH image is presented in Supplemental Fig. 1b.

In our cohort of TCC-UB, the 6p22.3 amplicon was amplified in 17% (62/364) of all patients (predominantly white), but with a much higher rate of 32% (12/38) in the much smaller subset of African Americans (p=0.0395) (Table 1). A comparison of 6p22.3 amplification in females (15%; 13/87) versus males (18%; 49/277) (t-test p=0.45), age <60 (19%; 15/81) versus age >60 (17%; 47/283) (p=0.96), and smokers (17%; 446/275) versus non-smokers (13%; 8/60) (p=0.68) showed no major differences. The results of 6p22.3 analysis with the standard pathological factors of tumor grade, depth of invasion (pT), and nodal metastatic disease (pN) supported that amplification is associated with a more aggressive phenotype. In regard of tumor invasion, there is a major difference in the rate of 6p22.3 amplification between superficial TCC-UB (9%; 13/137) and muscle-invasive TCC-UB (22%; 49/227) (p=7.2-04). Part of this difference is due to the near lack of 6p22.3 amplification in low-grade superficial TCC-UB (2%; 1/47) versus a much higher rate in high-grade superficial TCC-UB (13%; 12/93). An association of 6p22.3 amplification with tumor depth of invasion is also noted in muscle-invasive TCC-UB (p=0.12), where stage II showed 14% (7/51) amplified versus a rate of 24% (42/176) for stage III/IV. This marker of aggressive phenotype is also apparent in the evaluation of nodal metastatic disease as the rate of 6p22.3 amplification in pN>1 (32%; 27/85) was higher than for pN0 patients (16%; 18/112) (p=0.05). Interestingly, the rate of 6p22.3 amplification in pN>1 patients (40%; 19/48) was more than twice that in pN1 (21%; 8/38) patients (p=0.05).

**Table 1:** Clinicopathological Features of Patients with 6p22.3 Amplification

		325 White and 1 Asian				38 African American			
		Total		6p Amplified		Total		6p Amplified	
		#	%	#	%	#	%	#	%
Total Patients		326	100%	50	15%	38	100%	12	32%
Sex	Female	70	21%	8	11%	17	45%	5	29%
	Male	256	79%	42	16%	21	55%	7	33%
Age	<60	66	20%	9	14%	15	39%	6	40%
	>60	260	80%	41	16%	23	61%	6	26%
Smoking History	Current or previous smoker	268	82%	42	16%	7	18%	4	57%
	Never smoked	58	18%	8	14%	2	5%	0	0%
	Unknown	2	1%	0	0%	30	79%	8	27%
Bladder Cancer	Non-muscle invasive	118	36%	10	8%	19	50%	3	16%
	Muscle invasive	208	64%	40	19%	19	50%	9	47%
Superficial Bladder Cancer Grade	Low grade	40	12%	1	3%	7	18%	0	0%
	High grade	76	23%	9	12%	17	45%	3	18%
Superficial Bladder Cancer Stage	0a	62	19%	3	5%	11	29%	0	0%
	0is	2	1%	0	0%	1	3%	1	100%
	I	42	13%	5	12%	7	18%	2	29%
	IV	12	4%	2	17%	0			
Nodal Status MIBC	pN0	107	33%	16	15%	5	13%	2	40%
	pN1	35	11%	6	17%	3	8%	2	67%
	pN>1	41	13%	15	37%	6	16%	4	67%
	Unknown	0				5	13%	1	20%
Muscle Invasive Bladder Cancer Stage	II	45	14%	6	13%	6	16%	1	17%
	III	64	20%	13	20%	6	16%	2	33%
	IV	99	30%	21	21%	7	18%	6	86%
Outcome MIBC	Evidence Of This Cancer	98	30%	18	18%	4	11%	2	50%
	No Evidence Of This Cancer	96	29%	15	16%	4	11%	2	50%
	Unknown/Indeterminate	14	4%	6	43%	31	82%		

For the 181 muscle-invasive TCC-UB patients who underwent a cystectomy for curative intent, there was no significant association of amplification (35/181; 19.2%) with survival (log-rank p=0.438); However, the aggressive phenotype associated with high pT and pN stage resulted in more cases that were never disease-free (chi-square p=0.032, OR=2.65). In summary, these results indicate that 6p22.3 amplification is an early event in tumor progression. Although not associated with survival, it could potentially serve as a biomarker of extensive disease prior to cystectomy.

Among those with high grade superficial bladder cancers, African Americans had a higher percent with 6p22.3 amplification, compared to Whites (18% compared to 12%). African Americans, compared to Whites had a higher percent with the 6p22.3 amplification among those with both non-muscle invasive and muscle invasive disease. Among those with muscle invasive disease, African Americans, compared to Whites had a higher percent with the 6p22.3 amplification among those with muscle invasive cancer stage II (17% vs. 13%), III (33% vs. 20%), and IV (86% vs. 21%) and nodal stages pN0 (40% vs. 15%), pN1(67% vs. 17%), and pN>1(67% vs. 37%).

### Characterization of chromosomal 6p22 amplification in bladder cancer cell lines

**Figure 2:**
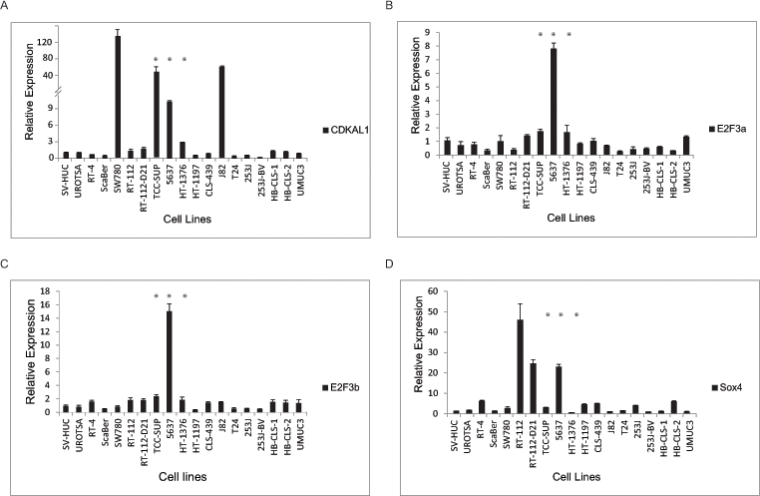
Candidate gene expression in bladder cancer cells CDKAL1 (A), E2F3a (B), E2F3b (C) and sox4 (D) expression by qRT-PCR in a panel of bladder cancer cell lines. E2F3, sox4 and CDKAL1 are highly expressed in chromosomal-6p22-amplicon-containing 5637 cells. (*, 6p22-amplicon-containing cell)

To further characterize the chromosomal 6p22 amplification, we performed FISH analysis in a collection of papillary bladder cancer cell lines as well as muscle-invasive bladder cancer cell lines. As a result, we identified three muscle-invasive bladder cancer cell lines (5637, TCC-SUP and HT1376) that contain amplification of the 6p22 region (Supplemental Table 2). We also performed qRT-PCR to test the gene expression levels in these bladder cancer cell lines. It was found that E2F3a, E2F3b, CDKAL1 and Sox4 were also highly expressed in the 6p22-amplified 5637 cells (Fig. 2), but the E2F3a and E2F3b mRNA levels in TCC-SUP and HT-1376 cells were similar to those in the control of non-6p22-amplifiedcells. We also noted gene expression values that were not correlated with the presence/absence of the chromosomal 6p22 amplification. For example, high expression of CDKAL1 was found in SW780 and J82 cells and high expression of Sox4 in RT-112 and RT-112-D21 cells, none of which contain the amplified 6p22 region.

**Figure 3:**
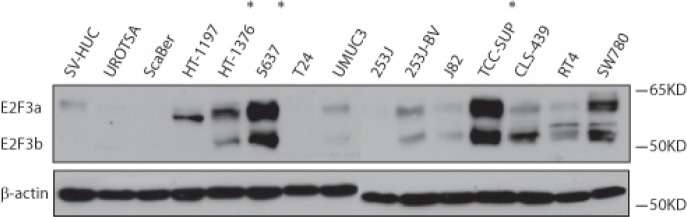
E2F3a and E2F3b protein expression in bladder cancer cells Increased E2F3a and E2F3b protein level as revealed by immunoblot in 6p22-amplicon-containing 5637 and TCC-SUP cells. (*, 6p22-amplicon-containing cell)

**Figure 4:**
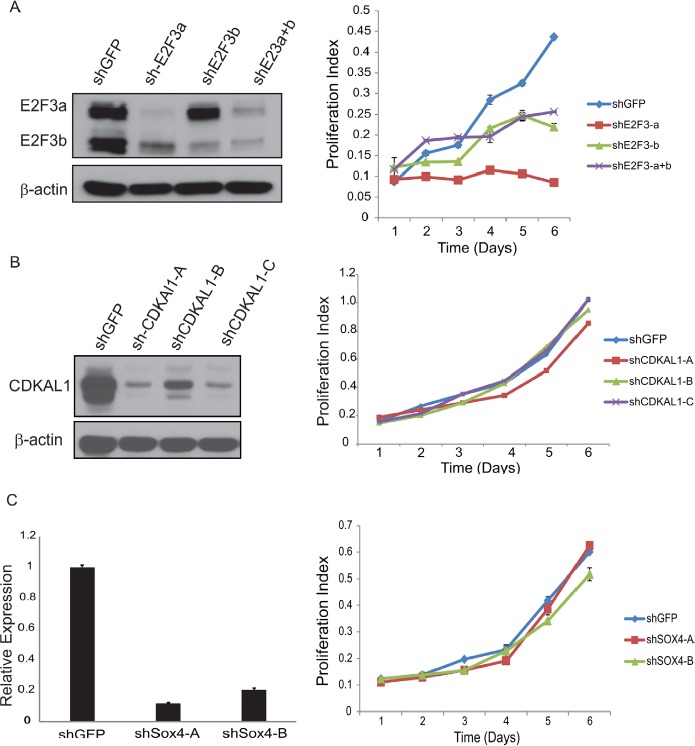
Knockdown of E2F3 inhibits cell proliferation A) Knockdown of E2F3 inhibits 5637 cell proliferation. Efficient knockdown of E2F3a, E2F3b and E2F3 as revealed by immunoblot (β-actin was used as a loading control) and cell proliferation examined by MTT assay. B) Knockdown of CDKAL1 has no effect on 5637 cell proliferation. Efficient knockdown of CDKAL1 as revealed by immnoblot (β-actin was used as a loading control) and cell proliferation examined by MTT assay. C) Knockdown of Sox4 has no effect on 5637 cell proliferation. Efficient knockdown of Sox4 as revealed by qRT-PCR and cell proliferation examined by MTT assay.

E2F3 belongs to the E2F transcription factor family. Most E2F proteins associate with a DP protein and form hetero-dimeric complexes that bind to DNA in a sequence specific manner [10]. E2F3 encodes two proteins (E2F3a and E2F3b) through the use of alternative promoters and different 5'-coding exons [11]. During G1/S, Rb protein becomes hyperphosphorylated, leading to the release of E2F3 and its target gene expression [12]. We first examined the E2F3 protein level in a panel of bladder cancer cell lines (Fig. 3). Indeed, we detected high level of E2F3 protein in the 6p22-amplified cells. To further investigate the role of E2F3 in cell proliferation, we designed specific shRNAs against E2F3a, E2F3b and both, and confirmed their knockdown efficiency by immunoblotting (Fig. 4a). Knockdown of E2F3a strongly inhibited cell proliferation, as well as the knockdown of E2F3b and both E2F3 isoforms (Fig. 4a). In contrast, knockdown of CDKAL1 and Sox4 in 5637 cells had no obvious effect on cell proliferation (Fig. 4b, c). To further confirm that the inhibited cell proliferation induced by E2F3 knockdown is dependent on chromosomal 6p22 amplification, we repeated the experiment in two other cell clines, 253J and T24, that do not contain the chromosomal 6p22 amplification (Supplemental Fig. 3). In contrast, knockdown of E2F3 failed to inhibit cell proliferation in these cell lines, indicating that the above role of E2F3 is dependent on the presence of chromosomal 6p22 amplification, and perhaps further through an “oncogene addiction” [13-15] mechanism.

### Identification of common and distinctive targets of E2F3

**Figure 5:**
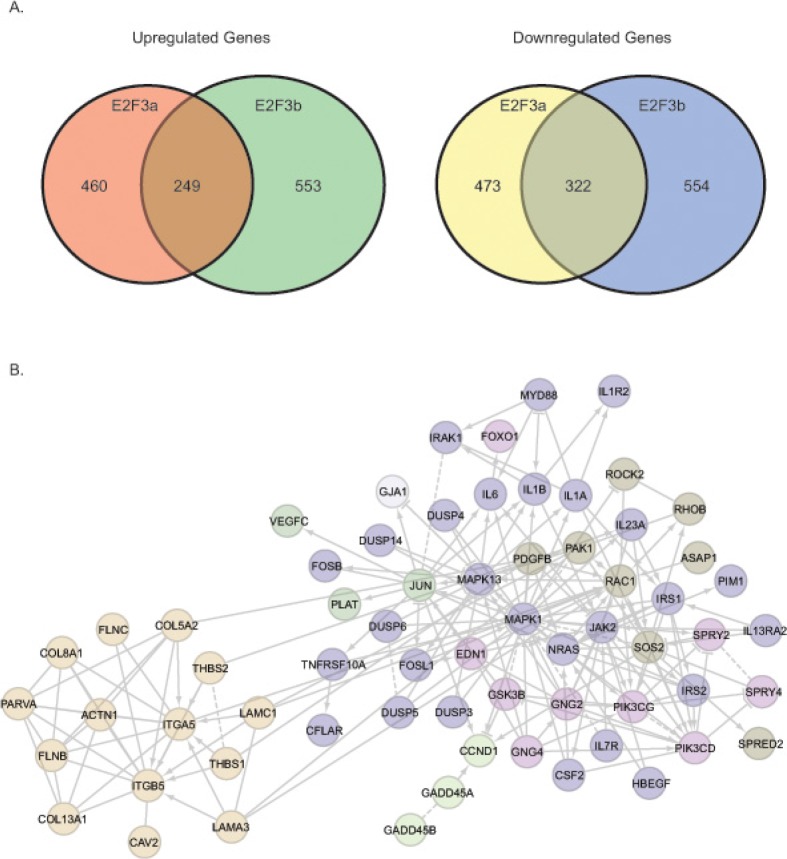
Gene expression profiling reveals common and distinctive E2F3a and E2F3b regulated genes A) Venn diagram showing common and unique up-regulated and down-regulated genes in response to knockdown of E2F3a or E2F3b. B) Signaling module components identified by integrative bioinformatics and pathway analyses in response to E2F3a knockdown.

To identify the specific genes regulated by E2F3a and E2F3b, we carried out a gene expression profile analysis using Illumina Human HT-12 v4 Expression BeadChips. We used 5637 cells treated with shGFP as a control and compared with those under the condition of E2F3a or E2F3b knockdown to reveal gene expression alterations. A 1.45-fold change and statistical value below 0.05 was used as cut-off in our analyses. Of note, we identified 249 genes that were up-regulated and 322 genes that were down-regulated in response to both E2F3a and E2F3b knockdown; 460 genes that were up-regulated and 473 genes that were down-regulated specifically upon knockdown of E2F3a; as well as 553 genes that were up-regulated and 554 genes that were down-regulated specifically upon knockdown of E2F3b (Fig. 5a; Supplemental Table 3).

We then focused on those genes that changed in response to the knockdown of E2F3a, as shE2F3a elicited a most pronounced phenotype. Among these candidate genes, we identified CCND1 as specifically down-regulated in response to shE2F3a. In addition, we found some signaling pathway changes in the presence of shE2F3a (Fig. 5b). For example, we detected changes of genes involved in the focal adhesion pathways, such as RAC1, ITGA5, ITGB5, COL5A2 and COL8A1; the interleukin and MAPK pathways, such as IL6, IL1A, IL1B, IL7R, MAPK1 and MAPK13; and the tyrosine receptor kinase pathways, such as HB –EGF, PI3KCD, PIK3CG, IRS1 and IRS2. Our gene expression profiling data demonstrated that E2F3 is not only involved in the classic cell cycle regulation, but also in other signaling pathways and cellular processes that regulate cell migration and invasion.

## DISCUSSION

In the present study, we found that chromosomal 6p22.3 amplification is specifically present in bladder cancer as compared to other cancer types in the TCGA dataset. In our cohort of TCC-UB, we found that chromosome 6p22.3 is frequently amplified in the muscle-invasive subset and has significant correlation to tumor stage. As a potential biomarker in TCC-UB, 6p22.3 amplification would provide risk assessment for advanced pT or pN stage in the pre-cystectomy biopsy setting where the only information provided in the typical surgical pathology report is presence or absence of muscle invasion. Given the current incurable status of advanced pT or pN stage TCC-UB and the significant morbidity and low rate of mortality associated with cystectomy, there is a clinical need to identify high-stage disease at the time of diagnosis.

Further gene expression profiling analyses revealed candidate oncogenes in a collection of bladder cancer cell lines. For example, proliferation advantage of the 5637 bladder cancer cell line is highly dependent on the 6p22.3 amplicon, in particular, the E2F3 gene within the locus, and knockdown of E2F3a or E2F3b significantly reduced cell proliferation. However, knockdown of Sox4 and CDKAL1 had minimal effect on cell proliferation [16-19]. Still, we could not rule out other potential functional effects that may result from the amplification of Sox4 and CDKAL1. For example, elevated Sox4 expression has been reported in a wide variety of tumors, including leukemia, colorectal cancer, lung cancer and breast cancer, suggesting a fundamental, yet not necessarily pro-proliferation, role in the development of these malignancies [20]. Indeed, in many cancers, deregulated expression of Sox4 has been associated with obscured tumor cell growth, survival, apoptosis, invasion or metastasis through the epithelial-to-mesenchymal transition (EMT) mechanism [20]. In addition, it was recently shown that sox4 potentiates the RAS-induced tumorigenesis in non-transformed breast cells and reduces the potential of highly metastatic derivatives of breast cancer [21]. Moreover, Tiwari et al recently demonstrated that Sox4 is a master regulator of epithelial-mesenchymal transition through controlling Ezh2 expression and epigenetic reprogramming [22]. Further studies are warranted to clarify the functional effects of Sox4 in 6p22.3-amplified TCC-UB.

“Oncogene addiction,” a term first coined in 2000 by Bernard Weinstein, reveals a possible “Achilles' heel” within the cancer cell that can be exploited therapeutically [13, 14]. In its simplest embodiment, oncogene addiction refers to the curious observation that a tumor cell, despite its plethora of genetic alterations, can seemingly exhibit dependence on a single oncogenic pathway or protein for its sustained proliferation and/or survival [15]. Our current study revealed that 6p22 is amplified in more than 20% of TCC-UB and represents a potential relatively common target in this tumor type. Given the factorial difficulty in targeting transcription factors, identification of the key downstream targets of E2F3 may serve as an intriguing therapeutic alternative. Of particular note, in our gene expression array analysis, we identified CCND1 as a potential target of E2F3. CCND1 is a key cell cycle regulator specifically responsible for the G1-to-S phase progression [23, 24]. Binding of cyclin D1 to cyclin-dependent kinase (CDKs) leads to the phosphorylation of retinoblastoma protein (pRb), and subsequently triggers the release of E2F transcription factors to allow the G1-to-S phase progression of the cell cycle. It will thus be of interest to test whether inhibition of CCND1 and CDK4/6 may reverse the oncogenic effect elicited by E2F3 amplification in TCC-UB with amplified 6p22.

We also found racial differences in the 6p22 amplification. At each stage African Americans had higher percentage with 6p22 amplification. This is very interesting because African Americans have a lower incidence of and higher mortality from bladder cancer compared to Whites [25-29]. Among this cohort African Americans were diagnosed with more aggressive disease and higher percent with 6p22 amplification. More research is needed to determine whether 6p22 amplification is associated with the reported racial differences in bladder cancer grade and stage of disease at diagnosis and survival outcomes [25-27, 29]. Furthermore in this cohort (at least in a subset of the cohort) African Americans were more likely to be smokers, more research is required to determine the interaction between smoking, 6p22 amplification and race with regards to bladder cancer aggressiveness and survival.

In summary, we have provided evidence that E2F3 in 6p22-amplified bladder cancer is a potential oncogene of importance. Future studies are needed to determine the interactions of E2F3 with other genes in the 6p22 amplicon such as SOX4. In the same manner, other potential oncogenes, such as CCND1, not in the 6p22 amplicon but yet frequently altered in bladder cancer, could have additional interactions.

## METHODS

### Patient Validation Cohort

Patients included in this study (total 335) were diagnosed and treated with transitional cell carcinoma of the urinary bladder (TCC-UB) between July 1970 and April 2011 at the Roswell Park Cancer Institute. Selection of patients included all in this time period with adequate material in the RPCI archival bank, i.e., adequate material for tissue microarray (TMA) construction and follow-up in the RPCI Tumor Registry. The RPCI Institutional Review Board gave approval for this study. The median patient age at first diagnosis was 69 years (average 68; range 36-91) with 263 males and 72 females. The majority (280 of 335; 81%) was either a current smoker at the time of diagnosis, or reported a history of smoking. The majority (325 of 335; 97%) were white, with the remainder of the cohort consisting of 9 African Americans and 1 American Indian. To more specifically describe this cohort of 335 patients, they were then divided into two general cohorts as either superficial TCC-UB (pTa, Tis, T1) or muscle-invasive TCC-UB (>pT2).

### Fluorescent In-situ Hybridization (FISH) for 6p Amplification

The 6p amplicon was evaluated by FISH using RP11 clones from the RPCI bacterial artificial chromosomal (BAC) library. A complete list of all BAC clones and probe design are listed in Supplementary Table 1. All FISH was done in a CLIA-certified NYS inspected laboratory using standard operating procedures on a manual platform. Specimens were evaluated with the Olympus BX51 microscope (Olympus Optical Company, LTD., Japan) under oil immersion at x100 magnification using the recommended filters. For each case the probe of interest was evaluated in at least 60 non-overlapping interphase tumor nuclei. Cells with no signals or with signals of only one color were disregarded. Tumors cells displaying at least 6 up to 10 signals of interest in one or more microclusters in the majority of tumors cells (>50%) were considered consistent with low-level amplification at that genomic coordinate. Tumors cells displaying >10 signals of interest in one or more microclusters in the majority of tumors cells (>50%) were considered consistent with high-level amplification at that genomic coordinate. Tumors cells displaying 6 or more intact signals but without microclusters in the majority of tumors cells (>50%) were considered consistent with polysomy and not amplification at that genomic coordinate. Cases with isolated large or bilobed cell tumor nuclei and increased signals of interest with or without microclusters were not considered as amplification as we consider these to be cells in mitosis with an extended chromatin pattern.

### Tissue Microarrays

Three 1-millimeter tissue cores from formalin-fixed paraffin embedded donor blocks were precisely arrayed into a new recipient paraffin block, including tumor specimens as well as controls. Specimens for controls within the TMA consisted of multiple cores of normal tissue from 10 different organs, including heart, colon, kidney, adrenal, ovary, myometrium, brain, thyroid, lung, and prostate, representing slightly more than 20% of all the cores in a TMA.

### Cell Culture

Human bladder cancer cell lines, 5637, TCC-SUP, HT-1376, 253J and T24 cells were cultured as described [19]. Transfection was performed using X-tremeGENE 9 DNA Transfection Reagent following the manufacturer's protocol (Roche). Packaging of lentivirus, cell transduction and drug selection were performed following standard protocols. For knockdown experiments, control shRNA was designed to target green fluorescent protein (GFP), a gene not expressed endogenously. shRNA hairpins targeting human E2F3a, E2F3b and E2F3 were cloned into pLKO.1 shRNA vector. The target sequences are listed (in the 5'-3' direction): shE2F3a: 5′-GCGTACATCCAGATCCTCACA-3′, shE2F3b 5′-GGAAATGCCCTTACAGCAGCA-3′, shE2F3 (a+b): 5′ AAGACCAAACTGTTATAGTTG-3′, shSOX4-A: 5'-AGCGACAAGATCCCTTTCATT-3', shSOX4-B: 5'-CCTTTCTACTTGTCGCTAAAT-3', shCDKAL1-D1: 5'-CCTGAACAGTTGCACTGTAAA-3', shCDKAL1-D2: 5'-CGATTGGATTTGCCGAAGATT-3', shCDKAL1-D3: 5'-AGTTGTGGAGGAGACAATTAA-3'.

### Real-time RT-PCR

For RNA preparation and qRT-PCR, RNA was extracted using the Trizol reagent (Invitrogen). cDNA synthesis was performed using the First-Strand cDNA Synthesis Kit (GE Healthcare) and quantitative real-time RT-PCR was performed using Power SYBR Green PCR Master Mix (Invitrogen). Sequences of the qPCR primer pairs (in the 5'-3' direction) are as follows: E2F3a-F: 5'-GCGCGTACATCCAGATCC-3', E2F3a-R: 5'-TTGGAGGGAGGAGGAACAG-3'; E2F3b-F: 5'-GCCCTTACAGCAGCAGGC-3', E2F3b-R: 5'-GGACTATCTGGACTTCG-3'; CDKAL1-F: 5'-TGGATTTGCCGAAGATTAG-3', CDKAL1-R: 5'-TTTCCTCTGGCGTGTTTAG-3'; SOX4-F: 5'-ACCGGGACCTGGATTTTAAC-3', SOX4-R: 5'-AAACCAGGTTGGAGATGCTG-3'; β-actin-F: 5'-CCAACCGCGAGAAGATGA-3', β-actin-R: 5'-CCAGAGGCGTACAGGGATAG-3'. Measurements were performed in triplicate and standardized to the levels of β-actin.

### Cell Proliferation (MTT) Assay

Five thousand to eight thousand bladder cancer cells were plated onto 96 wells plate. Plates were harvested daily. 20 ul of 5mg/ml MTT (3-(4,5-Dimethylthiazol-2-yl)-2,5-diphenyltetrazolium bromide, a tetrazole) were added to each well, incubated for 3.5 hours in 37 °C, and carefully removed without disturbing the cells. 150 ul MTT solvent (4 mM HCl, 0.1% Nondet P-40 (NP40), all in isopropanol was added into each well and plate shaken at room temperature for 15 min. Absorbance was read at 590 nm with a reference filter of 620nm.

### Amplification, labeling and BeadChip hybridization of RNA samples

The Illumina TotalPrep RNA Amplification Kit (Ambion) was used to transcribe 200ng of total RNA according to the manufacture's recommendation(s). Approximately, 700ng of cRNA was hybridized at 58°C for 16 hours to Illumina HumanHT-12 v4 Expression BeadChips (Illumina) and scanned using an Illumina BeadArray Reader and Bead Scan Software.

### Microarray data processing and pathway analysis

Data processing was performed with GenomeStudio version 2011.1 and the R Language and Environment for Statistical Computing (R) 2.13.2 in combination with Bioconductor 2.10 [30]. The Bioconductor lumi package [31] was used for quality control. The normalizations executed by Illumina GenomeStudio were all applied to the expression values on the original scale. If background adjustment was performed, we used the standard background correction offered by BeadStudio (bg_*); however, data was subsequently normalized using variance stabilization (VSN) and quantile normalization across samples using the functions in the ‘lumi’ Bioconductor (available at www.bioconductor.org). Differentially expressed genes were selected using a modified t-statistic with 15% of the standard deviation percentile as the fudge constant and fold change ratios, followed by Benjamini-Hochberg testing procedure for controlling FDR (False Discovery Rate) of 5% and permutation FDR of 7%, respectively. All methods used are implemented in the R packages lumi [31].

To investigate functional network and gene ontology relationships, candidate genes were first categorized using the Panther classification system [32]. For each molecular function, biological process or pathway term in PANTHER, the genes associated with that term were evaluated according to the likelihood that their numerical values were drawn randomly from the overall distribution of values. The Mann-Whitney U Test (Wilcoxon Rank-Sum Test) was used to determine the P-value relative to overall list of values that were input (nominal P value= 0.05).

Ingenuity Pathway Analysis (IPA) software was used to perform pathway analysis and describe functional relationships between gene products based on known interactions in the literature (http://www.ingenuity.com). Focus genes were overlaid onto a global molecular network developed from direct and/or indirect interactions, and sub-networks were algorithmically generated based on their connectivity. Pathways with a score greater than 4 (p<0.0001) were combined to form a composite network representing the underlying biological process.

### Statistical Analysis

Association between clinical/histological covariates and 6p22 amplification was tested using two-sample t-tests for the equality of proportions. Survival time associations were tested with a log-rank test. Statistical analysis of data was performed using the SPSS statistics software package (SPSS, IL). All results are expressed as mean ± SD.

## Supplemental Figures and Tables




