# Supplementary material for: 6p22.3 amplification as a biomarker and potential therapeutic target of advanced stage bladder cancer
Source: Oncotarget. 2013 Oct 29;4(11):2124–34. doi: 10.18632/oncotarget.1485 (PMC3875774; doi:10.18632/oncotarget.1485)
Supplement: Supplementary file 1 [file oncotarget-04-2124-s001.pdf]

6p22.3 Amplification as a Biomarker and potential therapeutic target of Advanced Stage Bladder Cancer - Shen et al

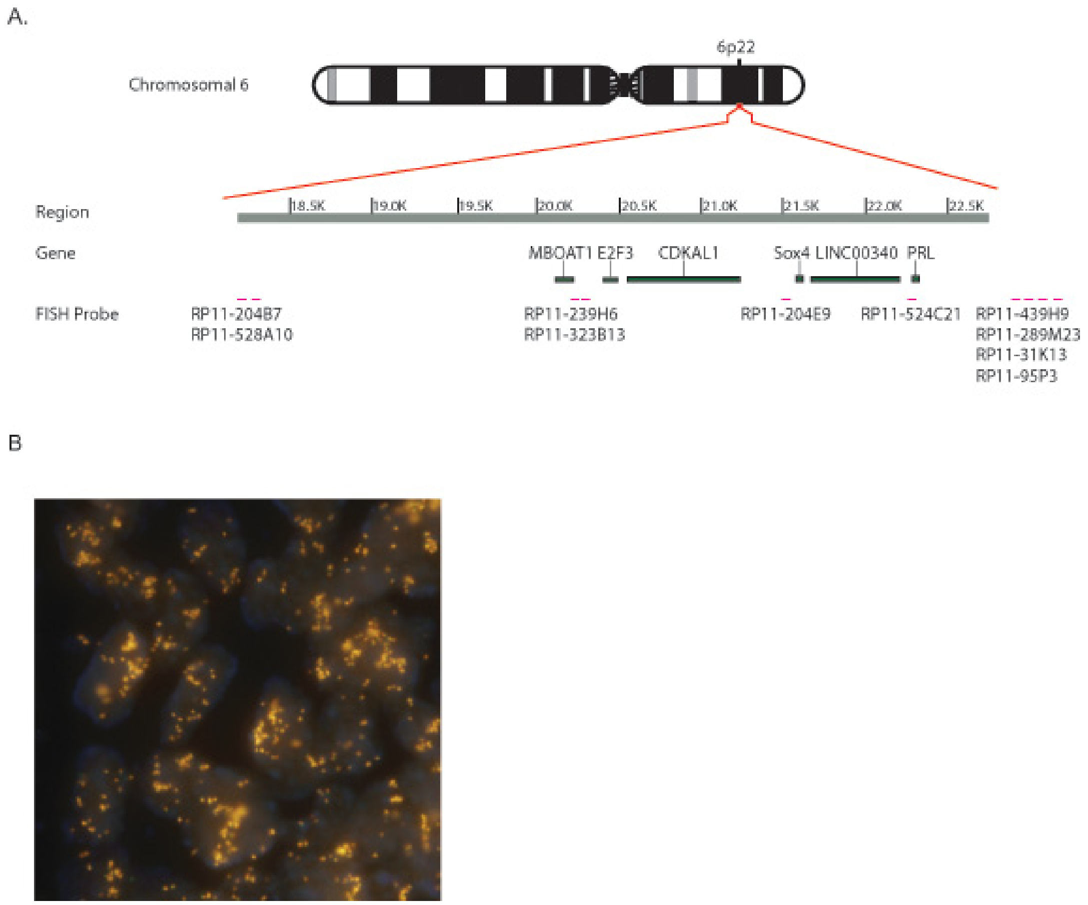

Supplemental Figure 1: A). Schematic illustration of FISH probes in the chromosome 6p22 region; B) Representative image of chromosomal 6p22 amplification under the microscope (X100).

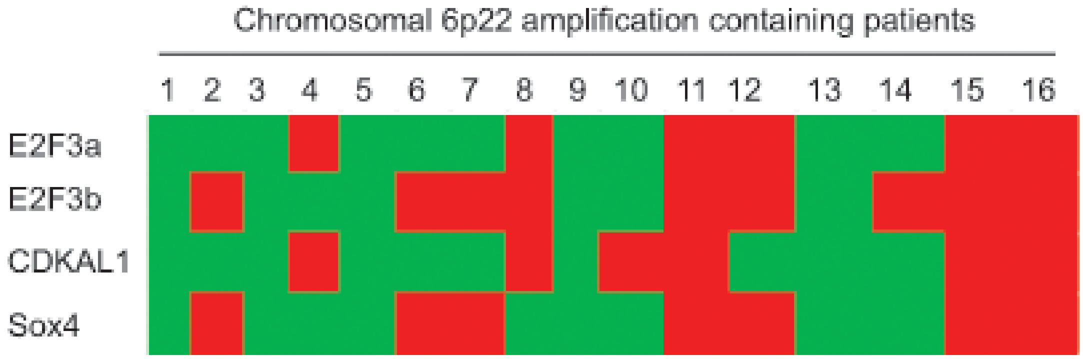

Supplemental Figure 2: RNA expression of *E2F3*, *sox4* and *CDKAL1* expression detected in 16 non-6p22–amplicon-containing and 16 6p22-containing tumor samples by qRT-PCR. Gene expressions of the16 non-6p22-amplicon samples are used as baseline. Red bar indicates gene expressions higher than baseline; green bar indicates gene expressions equal to or lower than base line.

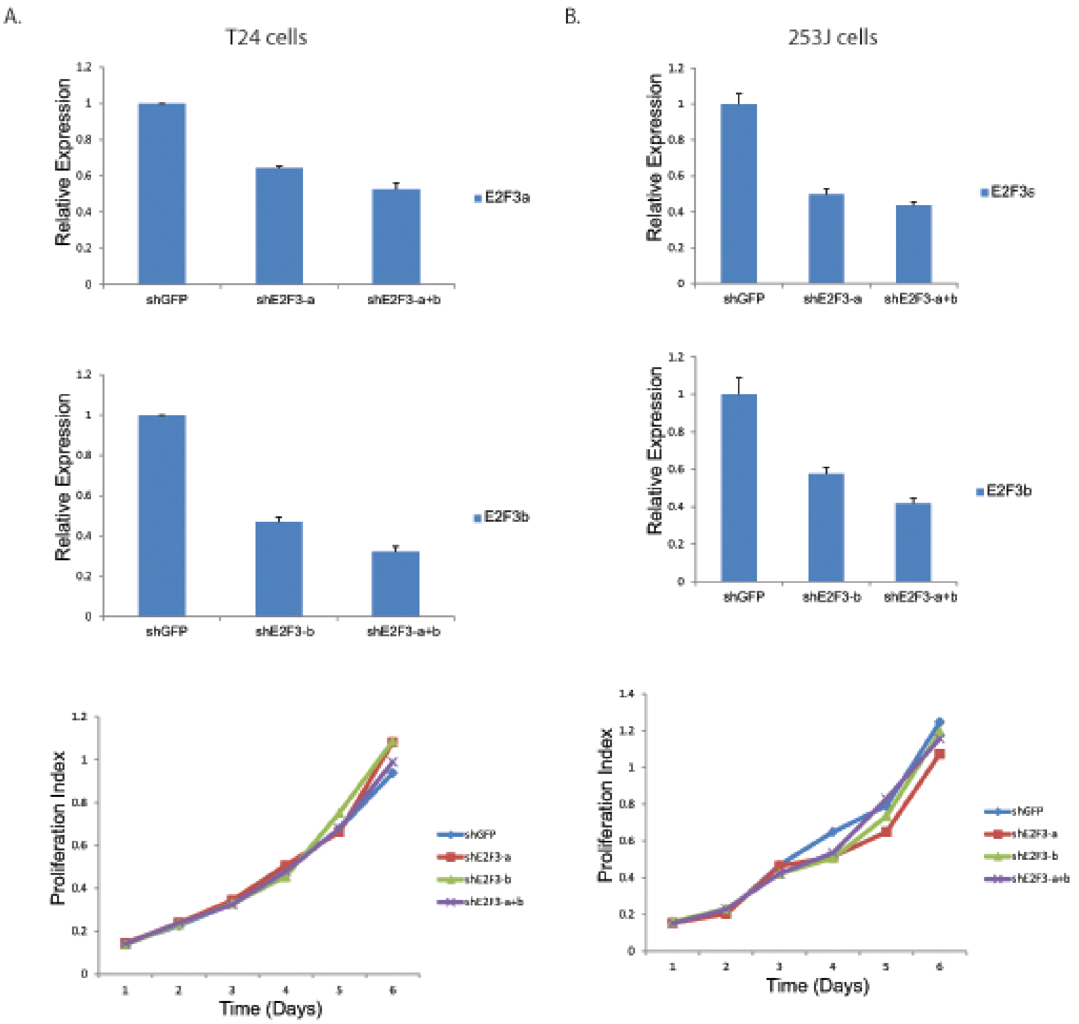

Supplemental Figure 3: Knockdown of E2F3 has no effect on proliferation of T24 (A) and 253J (B) cells that contain no 6p22 amplicon. Knockdown of *E2F3* as revealed by qRT-PCR and cell proliferation examined by MTT assay.
